# Forearm vs sternocleidomastoid autotransplantation after total parathyroidectomy for secondary hyperparathyroidism: a network meta-analysis and global bibliometric study

**DOI:** 10.3389/fendo.2026.1855948

**Published:** 2026-07-22

**Authors:** Yanjun Wang, Ruochen Bao, Fubo Zhang, Jiangtao Wang, Kai Cheng

**Affiliations:** 1 The Second Clinical Medical School of Shandong Medical and Pharmaceutical University, Yantai, China; 2 Department of Thyroid and Breast Surgery, Yantai Affiliated Hospital of Binzhou Medical University, Yantai, China

**Keywords:** autotransplantation, bibliometrics, forearm, network meta-analysis, secondary hyperparathyroidism, sternocleidomastoid muscle

## Abstract

**Background:**

Secondary hyperparathyroidism (SHPT) is a common complication in chronic kidney disease (CKD), and parathyroidectomy (PTX) remains the definitive treatment for refractory cases. However, the optimal surgical strategy, particularly regarding the implantation site for autotransplantation, remains uncertain. Total parathyroidectomy with autotransplantation (TPTX + AT) is widely performed, but it is unclear whether forearm autotransplantation (TPTX + ATF) offers advantages over autotransplantation into the sternocleidomastoid muscle (TPTX + ATSCM). Methods: Using the Web of Science Core Collection (WOSCC), we performed a bibliometric analysis. We then systematically searched PubMed, Embase, the Cochrane Library, and major Chinese databases (CNKI, Wanfang Data, and VIP) from inception to 1 November 2025. We included randomized controlled trials and cohort studies comparing subtotal PTX (SPTX), TPTX, TPTX + ATF, and TPTX + ATSCM. The primary outcomes were SHPT recurrence and postoperative hypocalcemia. Bayesian network meta-analysis (NMA) was conducted to rank the treatments.

**Results:**

Twenty-seven studies involving 6979 patients were included, comprising 2493 patients treated with SPTX, 964 with TPTX, 3359 with TPTX + ATF, and 163 with TPTX + ATSCM. Compared to TPTX, both SPTX and TPTX + ATF showed higher risks of recurrence (OR 3.80, 95% CrI 1.67–10.78; OR 2.66, 95% CrI 1.04–8.33). No significant differences were observed between TPTX + ATF and TPTX + ATSCM with respect to recurrence (OR 0.66, 95% CrI 0.13–3.05) or postoperative hypocalcemia (OR 1.18, 95% CrI 0.18–6.23). Bibliometric analysis revealed global research activity from the United States and China, with stable contributions over the past two decades.

**Conclusion:**

Forearm and sternocleidomastoid autotransplantation showed no statistically significant differences in recurrence or postoperative hypocalcemia based on the available evidence.Current evidence does not support a definitive preference for either implantation site, although forearm implantation may remain a reasonable option in settings where graft monitoring and potential reintervention are important considerations. High-quality randomized trials are still needed to confirm these findings.

**Systematic Review Registration:**

https://www.crd.york.ac.uk/PROSPERO/, identifier CRD420251236587.

## 1 Introduction

SHPT is a notable complication of CKD, with a prevalence of 30-50% in end-stage renal disease patients (1). Clinically, SHPT is characterized by persistently elevated parathyroid hormone (PTH) levels and parathyroid hyperplasia (2), which may lead to adverse outcomes such as persistent bone pain and increased mortality (3). The management of SHPT primarily includes dietary phosphate restriction, phosphate binders, correction of hypocalcemia, and vitamin D supplementation. While effective in most patients, medical therapy may fail or be poorly tolerated, in which case parathyroidectomy is strongly recommended (4, 5). Approximately 1-2% of patients with SHPT undergo PTX annually (6), and currently three main surgical procedures are used: SPTX, TPTX, TPTX + AT (7). The K/DOQI clinical practice guidelines for bone and mineral disorders in chronic kidney disease (5), as well as recommendations from most experts, endorse SPTX and TPTX + AT as preferred surgical options. However, the optimal surgical approach remains a subject of ongoing debate, and no standardized clinical guideline has yet been established.

TPTX aims to identify and remove all parathyroid tissue. However, concerns regarding persistent hypocalcemia, permanent hypoparathyroidism, and disturbances in bone metabolism have limited its application in clinical practice (6). SPTX involves the removal of three and a half parathyroid glands, with preservation of approximately 40 to 80 milligrams of the most normal-appearing parathyroid tissue left in place (8), but the residual parathyroid tissue may lead to a higher rate of recurrence, and reoperation in the neck becomes considerably more challenging once recurrence occurs. TPTX + AT involves removal of all parathyroid glands, followed by mincing the most normal-appearing gland into fragments measuring approximately 10 to 20 mm³ for autotransplantation. At present, the two most commonly performed forms of TPTX + AT are as follows: TPTX + ATF and TPTX + ATSCM (9). Since its introduction in 1968 (10), TPTX + AT has become the most widely used surgical approach in the majority of clinical treatment centers worldwide.

However, no widely accepted clinical guideline has established the optimal autotransplantation site after TPTX for SHPT, and no comprehensive network meta-analysis has systematically compared outcomes across implantation sites. Although the forearm and sternocleidomastoid muscle are the two most commonly used sites for autotransplantation (11, 12), the choice between them is still largely guided by institutional practice and surgeon preference rather than robust comparative evidence. The primary aim of this study was to compare the major surgical strategies used for SHPT with respect to recurrence and postoperative hypocalcemia. More specifically, we sought to determine whether forearm and sternocleidomastoid autotransplantation differ in these clinically relevant outcomes after TPTX. To this end, we performed a bibliometric analysis together with a systematic review and Bayesian network meta-analysis to characterize the current evidence base and provide a more integrated assessment of surgical decision-making in SHPT.

## 2 Materials and methods

### 2.1 Bibliometric analysis

Because the bibliometric analysis and the network meta-analysis served different objectives, they were conducted using separate search strategies, data sources, language restrictions, and time windows. In this study, we used the WOSCC as the primary information source and searched for publications on SHPT and parathyroid autotransplantation using terms related to “secondary hyperparathyroidism” and “autotransplantation.” (full search strategy in **Supplementary Content 1**). We restricted the search to publications written in English and limited the time frame from November 2006 through November 2025 to capture studies from the past two decades. To ensure the quality and relevance of the included literature, only articles and reviews were considered. We used VOSviewer (13) to display the major countries, institutions, authors, references, and their interrelationships, together with the corresponding visualization maps. In addition, we used the R package Bibliometrix (14) to further process the dataset and visualize the results. To minimize potential bias, all searches for the bibliometric analysis were performed on the same day.

### 2.2 Network meta-analysis

#### 2.1.1 Study protocol and registration

This systematic review and network meta-analysis was conducted in accordance with the Preferred Reporting Items for Systematic Reviews and Meta-Analyses PRISMA (version 2020) guidelines (15), (**Supplementary Content 2**) (16). The study protocol was prospectively registered in PROSPERO under the registration number CRD420251236587. And no AI was used in this research.

#### 2.2.2 Search strategy and study selection

Two experienced reviewers designed and executed the search strategy for the meta-analysis. We searched English language publications in PubMed, Embase, and the Cochrane Library, and Chinese language publications in CNKI, Wanfang Data, and VIP databases. The search covered all records from the inception of each database to November 1, 2025. During revision, we repeated the database search using the same predefined strategy and manually re-checked the reference lists of the included studies and relevant reviews to identify potentially missing eligible publications. All potentially relevant records identified during this verification process were reassessed against the prespecified eligibility criteria by two reviewers, with disagreements resolved by a third reviewer. The search strategy combined Medical Subject Headings terms with free text words for a comprehensive search. The search algorithm was constructed using combinations of the terms presented in **Supplementary Content 3**.

#### 2.2.3 Inclusion and exclusion criteria

The inclusion criteria for relevant studies were as follows: 1) randomized controlled trials (RCTs) or prospective or retrospective cohort studies that evaluated two or three treatment strategies, including TPTX, SPTX, TPTX + ATF and TPTX + ATSCM; 2) Comparison of the short-term and long-term efficacy of TPTX, SPTX, TPTX + ATF, and TPTX + ATSCM in patients with SHPT; 3) The clinical outcomes of the included studies were required to contain at least one of the following endpoints: recurrence or postoperative hypocalcemia.

The exclusion criteria were: 1) studies involving primary or tertiary hyperparathyroidism in which data specific to renal SHPT could not be separately extracted; 2) articles categorized as letters, case reports, reviews, commentaries, editorials, or conference abstracts; 3) studies that evaluated zero or only one eligible surgical strategy, had fewer than ten participants, or lacked extractable data for recurrence or postoperative hypocalcemia; and 4) studies in which overlapping populations were reported without additional outcome data relevant to the prespecified comparisons.

#### 2.2.4 Data extraction and quality assessment

Two researchers independently reviewed and screened all potentially eligible articles. Any disagreements were resolved through discussion with a third reviewer. The following information was extracted in a standardized format: name of the first author, year of publication, study location, study design, study period, surgical procedure, participant characteristics (including sample size and age), and duration of follow-up. For randomized controlled trials, we assessed methodological quality using Review Manager version 5.4.1 developed by the Cochrane Collaboration. For non-randomized cohort studies, quality assessment was performed using the Newcastle–Ottawa Scale (NOS).

In this study, each of the three domains was evaluated independently, and one star was awarded for each item that met the predefined criteria. Quality assessment was performed by two independent reviewers, with a third reviewer consulted to resolve any uncertainties. Studies with a score of five or higher were considered to have a low risk of bias. Publication bias was assessed using funnel plots, and asymmetry in the funnel plot was interpreted as indicative of potential publication bias.

Recurrence and postoperative hypocalcemia were extracted according to the definitions reported in the original studies. When available, we also recorded the timing and clinical or biochemical criteria used for outcome assessment. Because these definitions were not fully uniform across studies, this variability was considered when interpreting the pooled results.

#### 2.2.5 Statistical analysis

NMA and the associated graphical analyses were conducted in R software version 4.5.0 (The R Project for Statistical Computing) using the “gemtc” and “rjags” packages (17, 18). In the patient-based NMA model, we selected a random effects model with four chains, 5,000 burn in iterations, 10,000 sampling iterations, and a thinning interval of one. These settings were chosen to sufficiently eliminate the influence of initial values, increase the number of iterations and thinning steps, and minimize Markov chain Monte Carlo error and variation in the deviance information criterion, yielding highly stable graphical outputs (19). Consistency was assessed by examining agreement between direct and indirect treatment effects, and a node splitting approach was applied to detect potential inconsistency in any treatment comparison within the network (20). Funnel plots were used to evaluate publication bias. Treatment effects were summarized using odds ratios (ORs) and 95% credible intervals (CrIs), and the results were presented using network plots and forest plots. Rankograms and Surface Under the Cumulative Ranking Curve (SUCRA) were used to provide a comprehensive ranking of the surgical strategies for postoperative recurrence and hypocalcemia; higher SUCRA values indicate a higher probability of being ranked as the worst treatment for a given outcome.

## 3 Results

### 3.1 Bibliometric analysis

**Figure 1:**
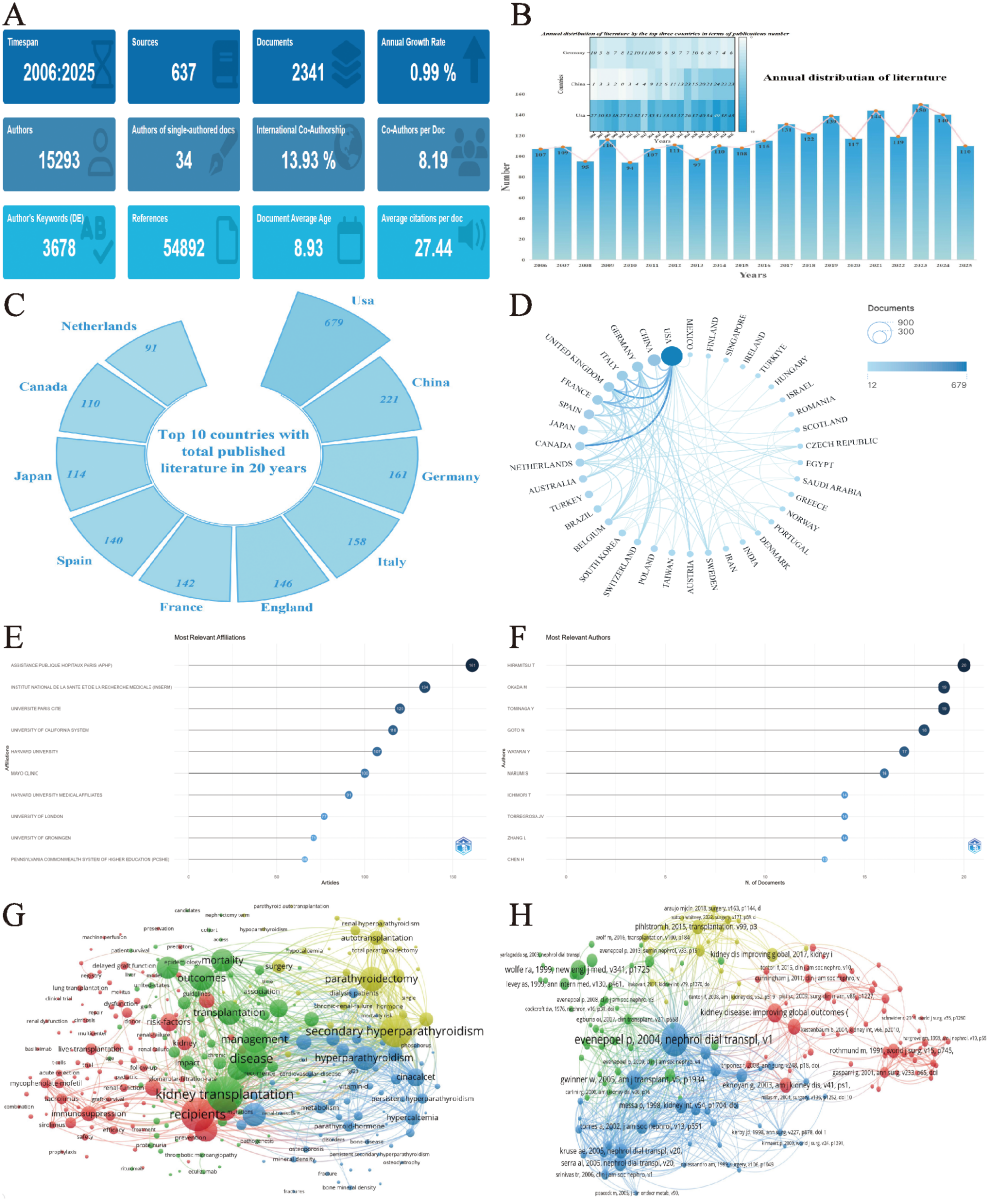
Bibliometric analysis of literature on SHPT and autotransplantation from 2006 to 2025. **(A)** The overview of SHPT and autotransplantation in WOSCC SCI-EXPANDED by ‘bibiometrix’ package. **(B)** The total number of publications published each year from 2006 to 2025 and the annual distribution of literature in the top three countries with the number of publications. **(C)** Top 10 countries with total published literature from 2006 to 2025. **(D)** Global country cooperation network map from 2006 to 2025. **(E)** The top 10 most relevant affiliations on SHPT and autotransplantation from 2006 to 2025. **(F)** The top 10 most relevant authors on SHPT and autotransplantation from 2006 to 2025. **(G)** VOSviewer visualization map of co-occurrence authors in SHPT and autotransplantation from 2006 to 2025. **(H)** VOSviewer visualization map of co-citation cited references in SHPT and autotransplantation from 2006 to 2025.

We identified a total of 2341 relevant reviews and research articles. Between 2006 and 2025, 637 journals and 15,293 authors contributed to the published literature. In total, 3678 keywords were extracted, and 54,892 references were cited. These findings indicate that SHPT and parathyroid autotransplantation have remained major global research hotspots over the past two decades (**Figure 1A**). The overall annual publication output in this field, together with the annual distribution of publications from the three leading countries, provides a general temporal overview rather than a technique-specific analysis of surgical procedures (**Figure 1B**). Interestingly, although the overall volume of research has remained relatively stable, notable differences were observed in the contributions of individual countries. The United States produced the largest number of publications, with 679 articles, maintaining a leading position in this field, followed by China with 221 articles and Germany with 161 articles (**Figure 1C**). Since 2017, China has demonstrated a rapid increase in publication output (**Figure 1B**). Notably, the United States leads this field in publication output and international collaboration, with strong collaborative networks also involving Canada, the United Kingdom, China, France, and Germany (**Figure 1D**). In addition, the Assistance Publique – Hôpitaux de Paris and the Institut National de la Santé et de la Recherche Médicale are the two most academically influential institutions in this field, indicating the significant contributions of French research institutions to the advancement of SHPT and parathyroid autotransplantation research (**Figure 1E**). We also found that Takahisa Hiramitsu and Manabu Okada are the most productive and influential authors in this field, and their contributions have had a substantial impact on the research and practical applications of SHPT and parathyroid autotransplantation (**Figure 1F**).

In summary, bibliometric analysis indicates sustained global research interest in SHPT and parathyroid autotransplantation, providing a robust foundation for comparative evaluation. Accordingly, we performed a network meta-analysis to compare key outcomes across different TPTX + AT, offering conclusive guidance for clinical practice.

These bibliometric findings underscore the sustained global interest in surgical management of SHPT and provide a strong academic basis for a comparative evaluation of surgical strategies, which we addressed using a NMA.

### 3.2 Network meta-analysis

#### 3.2.1 Study selection

**Figure 2:**
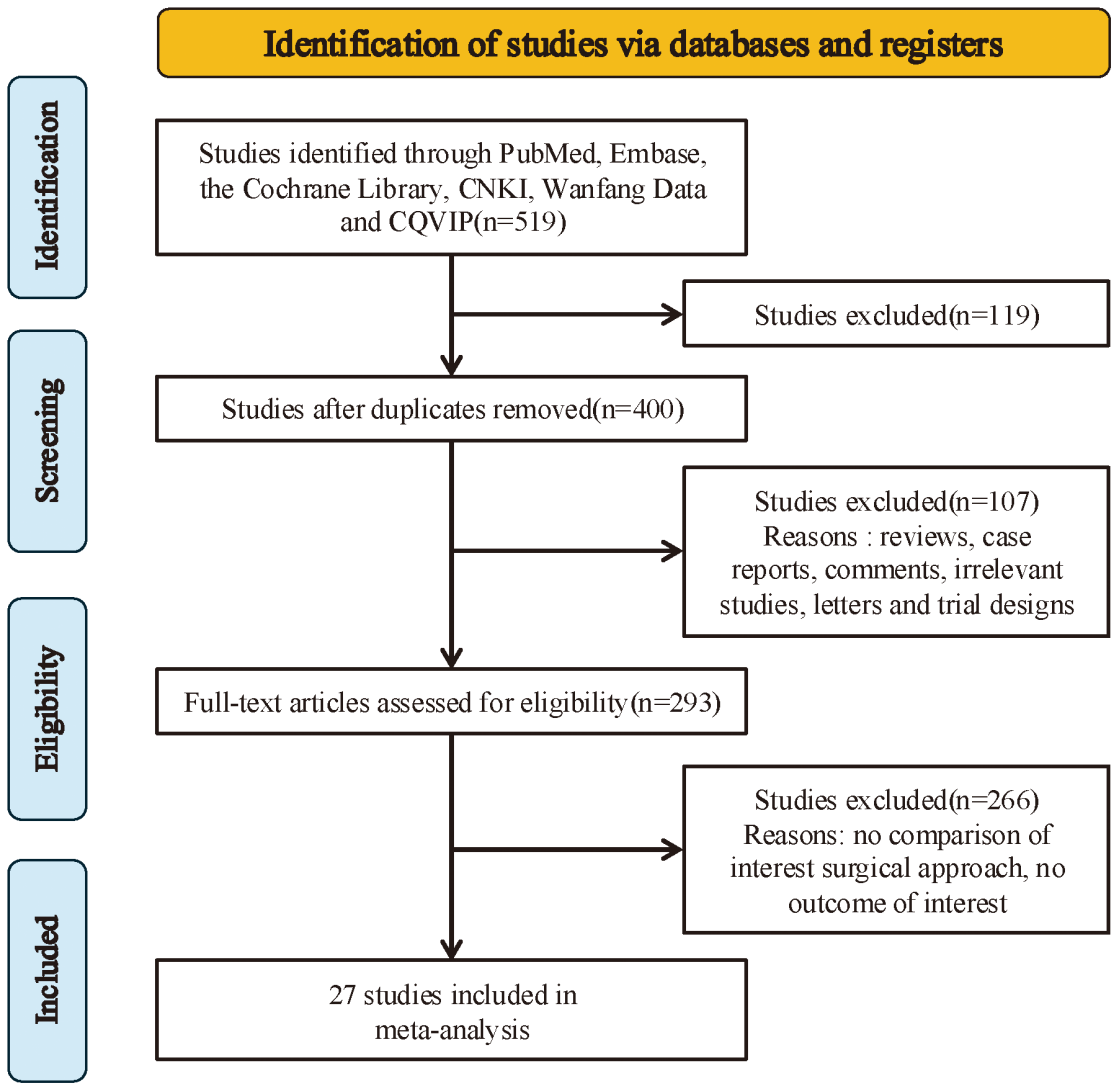
Flow diagram of the identification process of the eligible studie.

A total of 591 potentially relevant articles were initially screened. After removing duplicates, 400 studies were retained for further review. Following an examination of titles and abstracts, 373 studies were excluded due to failure to meet the inclusion criteria. Ultimately, 27 studies were included in this analysis (21–47). Articles that appeared potentially relevant but were not included were excluded mainly because they were single-arm surgical series, did not compare at least two eligible strategies, addressed non-renal hyperparathyroidism without separately extractable SHPT data, or lacked extractable outcome data. Although the database search for the network meta-analysis was updated to 1 November 2025, the studies ultimately included were published between 1973 and 2022, comparing the therapeutic effects of four different surgical approaches for patients with SHPT: TPTX, SPTX, TPTX + ATF, and TPTX + ATSCM. The TPTX group included 964 patients, the SPTX group included 2493 patients, the TPTX + ATF group included 3359 patients, and the TPTX + ATSCM group included 163 patients. The relatively small number of patients in the TPTX + ATSCM group reflects the distribution of the eligible comparative literature in this network meta-analysis and should not be taken as direct evidence of reduced contemporary use of this approach. A total of 20 retrospective cohort studies, 5 prospective cohort studies, and 2 randomized controlled trials were included. The follow-up duration ranged from 6 months to 8 years. The sample sizes of the included studies ranged from 15 to 898 participants. The study selection process is presented in **Figure 2**. In addition, 22 of the included studies were two-arm trials, whereas 5 studies employed a three-arm design involving three different treatment strategies.

#### 3.2.2 Quality assessment and publication bias

**Figure 3:**
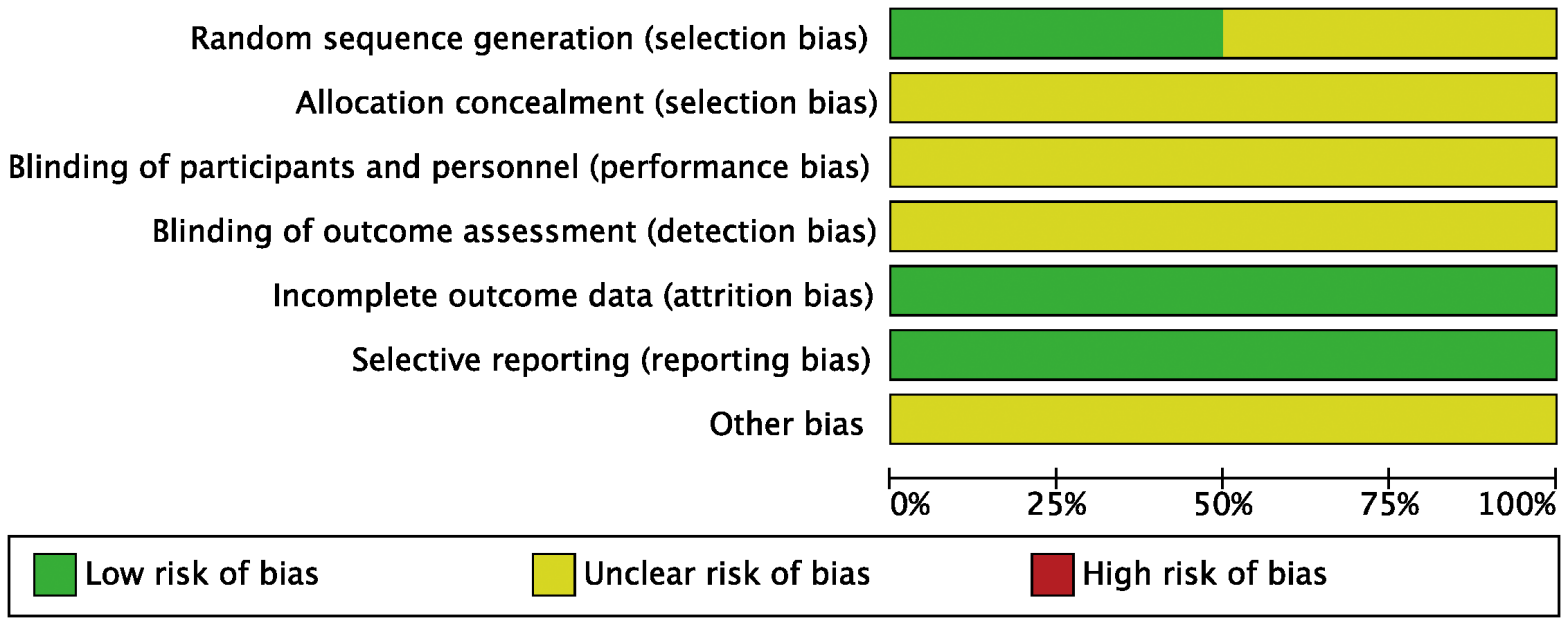
Risk of bias graph: review authors’ judgements about each risk of bias item presented as percentages across all included studies.

**Figure 4:**
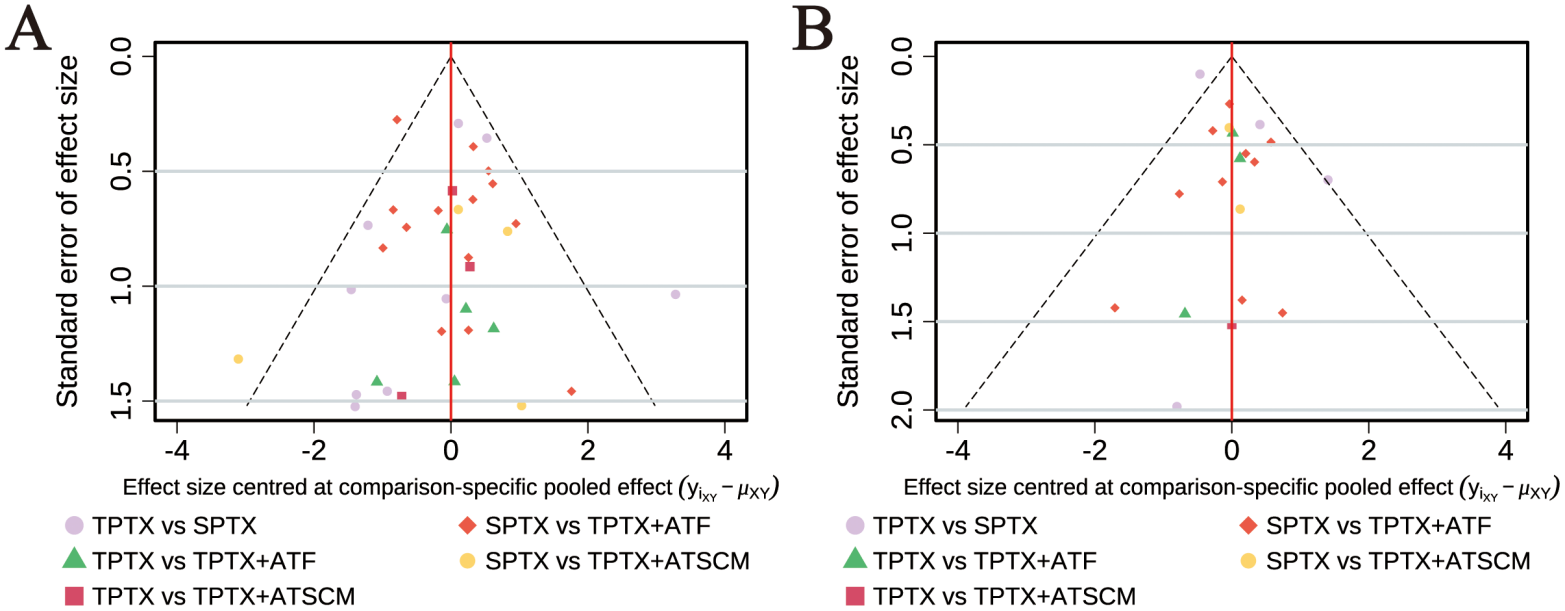
Funnel plot of this network meta-analysis, with different colors representing different comparisons. **(A)** Funnel plot for recurrence; **(B)** Funnel plot for postoperative hypocalcemia.

The baseline characteristics and methodological assessments of the included studies are summarized in **Table 1**. To facilitate comparison across studies, **Table 1** provides an integrated overview of the first author and publication year, country, study design, study period, surgical strategy, sample size, mean age, follow-up duration, and NOS assessment, including selection, comparability, outcome, and total score. This summary allows readers to evaluate the clinical and methodological characteristics of the included evidence more directly, particularly with respect to study design, operative method, sample size distribution, follow-up duration, and study quality. The Cochrane risk-of-bias evaluations for the randomized controlled trials meeting the inclusion criteria are shown in **Figure 3**. The overall quality of the 27 included studies was considered satisfactory. In the NMA model for the outcome of recurrence, most individual studies were evenly distributed on both sides of the inverted funnel plot, indicating a low likelihood of publication bias (**Figure 4**).

**Table 1:** Characteristics and NOS assessments of the included studies.

Study (year)	Country	Study design	Study period	Operative method	Sample Size	Mean age	Follow-up	Newcastle-ottawa scale			
								Selection	Comparability	Outcome	Score
Y. Liang 2015	China	PCS	2010-2014	SPTX	21	53.2 ± 12.7	6 months	★★★★	★★	★★★	9
				TPTX+ATF	21						
				TPTX	21						
Radu Mircea Neagoe 2016 (22)	Romania	PCS	2010-2015	SPTX	26	50.0 ± 10.6	18 months	★★★★	★	★★★	8
				TPTX+AT SCM	19	51.1 ± 11.1					
Gaylene M. Hargrove 1999 (23)	Canada	RCT	1992-1996	SPTX	28	–	22-28 months	–	–	–	–
				TPTX+ATF	8						
Kevin Anderson Jr 2017 (24)	United States	RCS	2005-2013	SPTX	765	48 (37–58)	–	★★★★	★	★★★	8
				TPTX+ATF	365	47 (37-56)					
Gürhan Sakman 2014 (25)	Turkey	RCS	2003-2011	SPTX	25	42.6 ± 14.5	65 months	★★★★	★★	★★★	9
				TPTX+ATF	25	36.4 ± 11.7					
Tsu-Hui (Hubert) Low 2009 (26)	Australia	RCS	1988-2004	SPTX	68	20.1-75.6	–	★★★	★	★★★	7
				TPTX+AT SCM	4						
				TPTX	43						
Polina V Zmijewski 2019 (27)	United States	RCS	2006-2017	SPTX	23	51.7~15.1	>6 months	★★★	★	★★★	7
				TPTX+AT SCM	23	48.4~11.5					
Ralph Schneider 2011 (28)	Germany	RCS	1976-2009	SPTX	21	48.5 ± 0.57	6 months	★★★	★	★★★	7
				TPTX+ATF	504						
				TPTX	32						
Claudio Casella 2023 (29)	Italy	RCS	2007-2020	SPTX	14	54 (26–75)	24 months	★★★★	★★	★★★	9
				TPTX+ATF	38	56.5 (14–73)					
Parameswaran Rajeev 2016 (30)	Singapore	PCS	2006-2013	SPTX	24	50 ± 7	24.6–81.8 months	★★★★	★	★★★	8
				TPTX+ATF	57	54 ± 8					
Wenqiang Qiu 2023 (31)	China	RCS	2014-2022	SPTX	20	46.32 ± 11.81	–	★★★	★	★★	6
				TPTX+ATF	60						
Radu Mircea Neagoe 2021 (32)	Romania	PCS	2010-2017	SPTX	29	51.45 ± 10.5	>18 months	★★★★	★	★★★	8
				TPTX+AT SCM	33	51.3 ± 10.9					
				TPTX	15	56.5 ± 7.14					
Ramazan Sari 2019 (33)	Turkey	RCS	2012-2018	SPTX	37	43.1 ± 16.7	37.5(36) months	★★★	★	★	5
				TPTX+ATF	25	41.4 ± 15.8	41(28) months				
Matthias Rothmund 1991 (34)	Germany	RCT	1984-1985	SPTX	20	44.8	–	–	–	–	–
				TPTX+AT F	20	45					
Yoshihiro Tominaga 1992 (35)	Japan	RCS	1973-1991	SPTX	19	44.6	6 months	★★★	★	★	5
				TPTX+ATF	229						
Stefan Ockertc 2002 (36)	Germany	RCS	1993-1999	TPTX+ATF	11	–	23 months	★★★	★	★★	6
				TPTX	11						
P. Nichols 1990 (37)	Canada	RCS	1978-1987	SPTX	34	41.2	–	★★★	★	★★	6
				TPTX+ATF	39	41.6					6
Susumu Matsuoka 2007 (38)	Japan	RCS	1973-2005	SPTX	20	52.1-10.9	6-261 months	★★★	★	★★	
				TPTX+ATF	1837						
Qingqing He 2014 (39)	China	RCS	2008-2012	TPTX+AT SCM	14	45.8 ± 14.2	42 months	★★★	★	★★★	7
				TPTX	33	46.5 ± 13.9					
ShuYong Zhang 2019 (40)	China	RCS	2016-2018	TPTX+AT SCM	70	25-70	22 months	★★★★	★	★★★	8
				TPTX	54						
HengYuan Gao 2017 (41)	China	RCS	2015-2016	SPTX	56	50(25-70)	6-18 months	★★★★	★	★★★	8
				TPTX	54						
Nada Rayes 2008 (42)	Germany	RCS	1997-2006	SPTX	16	–	31(10-97) months	★★	★	★★	5
				TPTX	17		41(15-78) months				
C. Dotzenrath 1995 (43)	Germany	PCS	1985-1995	SPTX	89	–	8 years	★★★	★	★★	6
				TPTX+ATF	54						
ChanYuan Yu 2017 (44)	China	RCS	2014-2016	SPTX	40	46.35 ± 11.97	6 months	★★★★	★	★★★	7
				TPTX+ATF	16						
				TPTX	16						
Lindsay E. Kuo 2014 (45)	United States	RCS	2008-2011	SPTX	662	49	–	★★★	★	★★	6
				TPTX	236						
Elin Isaksson 2019 (46)	Sweden	RCS	1991-2013	SPTX	436	60 (14)	6.0 (5.0) months	★★★	★	★★	6
				TPTX	388	61 (13)	6.9(4.9) months				
Ming-Lang Shih 2008 (47)	China	RCS	1995-2006	TPTX	44		41.3 months	★★★	★	★★	6
				TPTX+ATF	50						

RCS: retrospective cohort study; PCS: prospective cohort study; RCT: randomized controlled trial.

★ indicate the NOS quality score and the total number of asterisks equals the overall NOS score for each study.

**Table 1** legend: The table includes the following information for each study: first author and publication year, country, study design, study period, operative method, sample size, mean age, follow-up duration, and Newcastle–Ottawa Scale assessment, including selection, comparability, outcome, and total score.

#### 3.2.3 Evidence network

**Figure 5:**
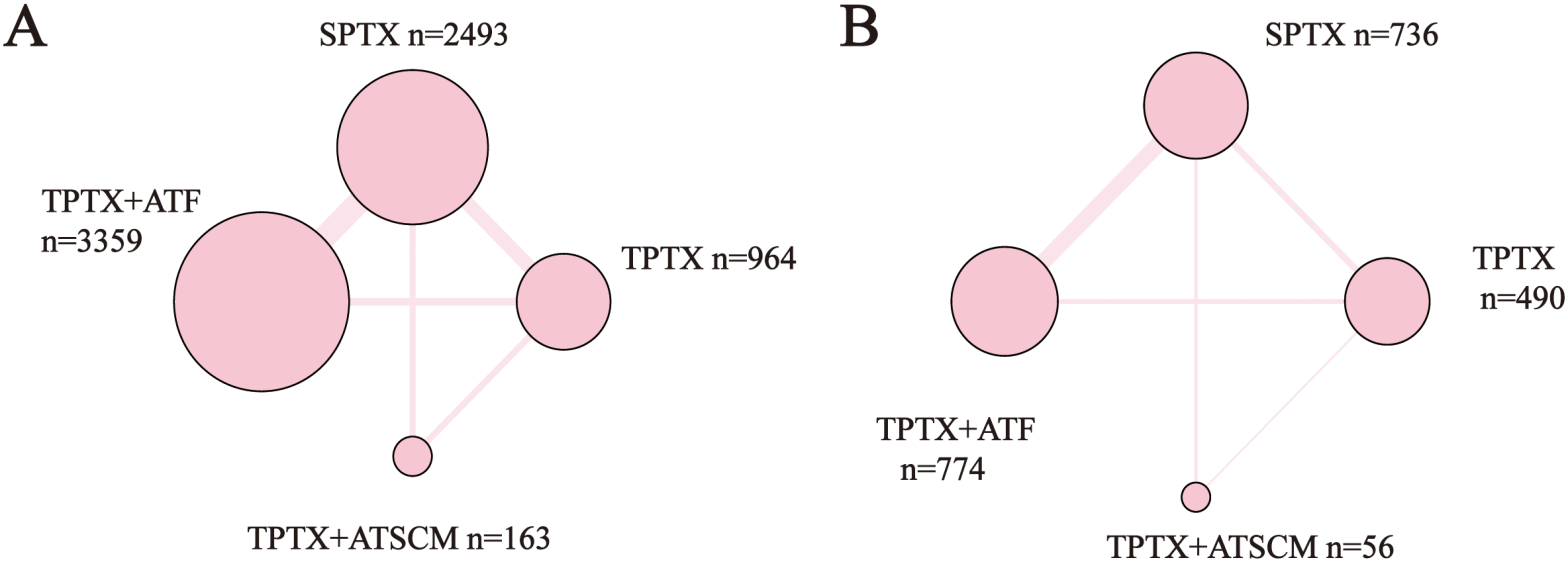
Network of eligible comparisons. The width of the lines is proportional to the number of clinical trials comparing each treatment pair. The size of each node corresponds to the number of randomly assigned participants (sample size). **(A)** Network of eligible comparisons for recurrence; **(B)** Network of eligible comparisons for postoperative hypocalcemia.

In the network plot evaluating recurrence for the different surgical approaches to SHPT, the size of each node represents the sample size or study weight, and the width of each connecting line represents the number of direct comparative studies, thereby providing a visual depiction of the direct relationships and closed loops among the interventions. The results show that studies involving TPTX + ATF and SPTX included larger numbers of patients, received greater research attention in relation to recurrence, and were supported by a higher number of direct comparative trials (**Figure 5**).

#### 3.2.4 Test for inconsistency

The node-splitting method was employed to evaluate the agreement between direct and indirect evidence. The results demonstrated consistency across all outcomes; consequently, a consistency model was adopted for this study (P > 0.05).

#### 3.2.5 Network meta-analysis results

**Figure 6:**
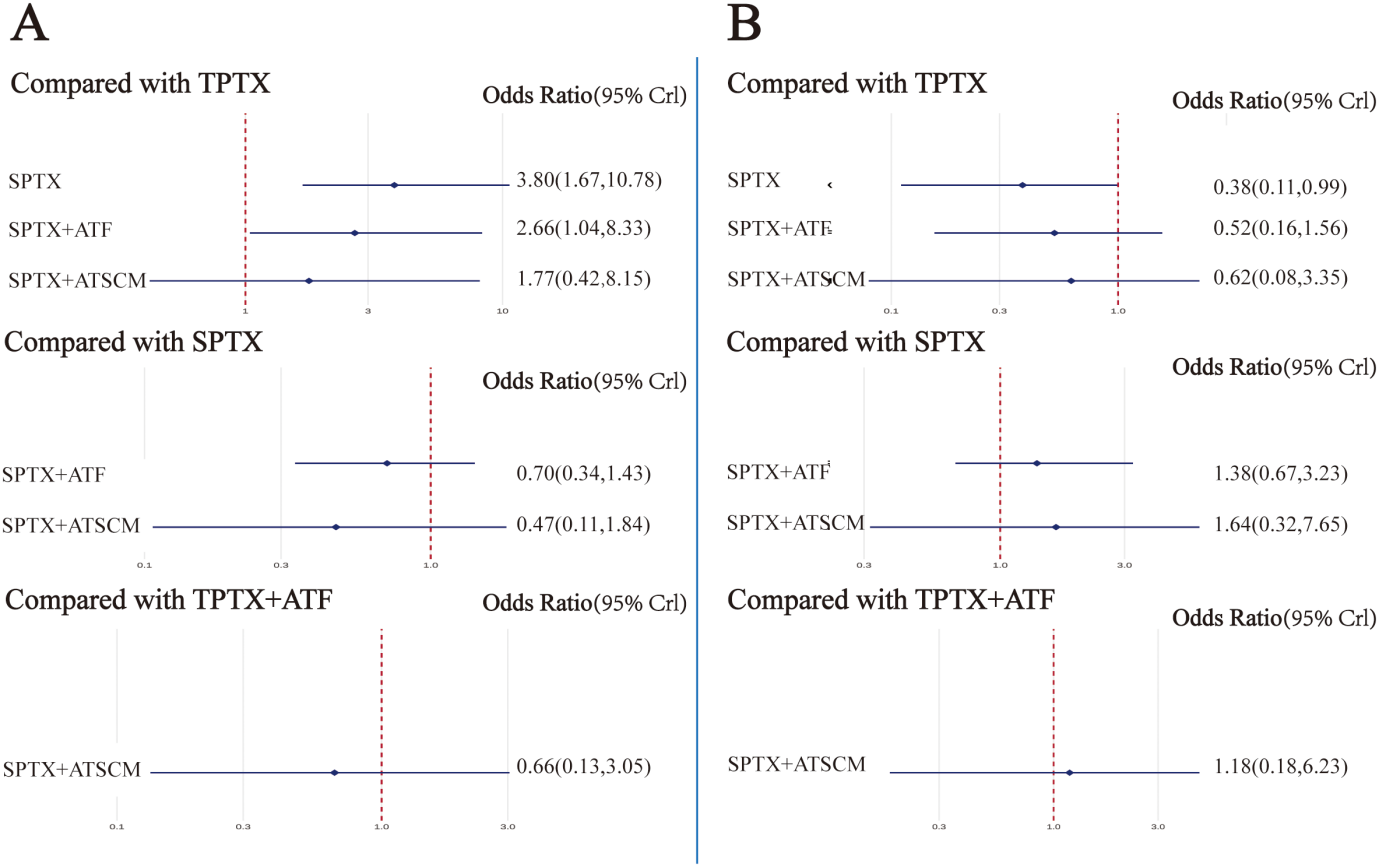
Forest plot of eligible comparisons. The results were evaluated by the odds ratio (OR) with a 95% confidential interval (CrI). **(A)** Forest plot of network meta-analysis for recurrence; **(B)** Forest plot of network meta-analysis for postoperative hypocalcemia.

A total of 27 studies (enrolling 6,979 participants) were included in the analysis of recurrence. Compared with TPTX, both SPTX and TPTX + ATF significantly increased the recurrence rate of postoperative SHPT (OR 3.80, 95% Crl 1.67*–*10.78; OR 2.66, 95% Crl 1.04*–*8.33). However, no statistically significant differences were observed when comparing TPTX + ATF versus SPTX (OR 0.70, 95% Crl 0.34*–*1.43), TPTX + ATSCM versus SPTX (OR 0.47, 95% Crl 0.11*–*1.84), or TPTX + ATSCM versus TPTX + ATF (OR 0.66, 95% Crl 0.13*–*3.05) (**Figure 6A**).

**Figure 7:**
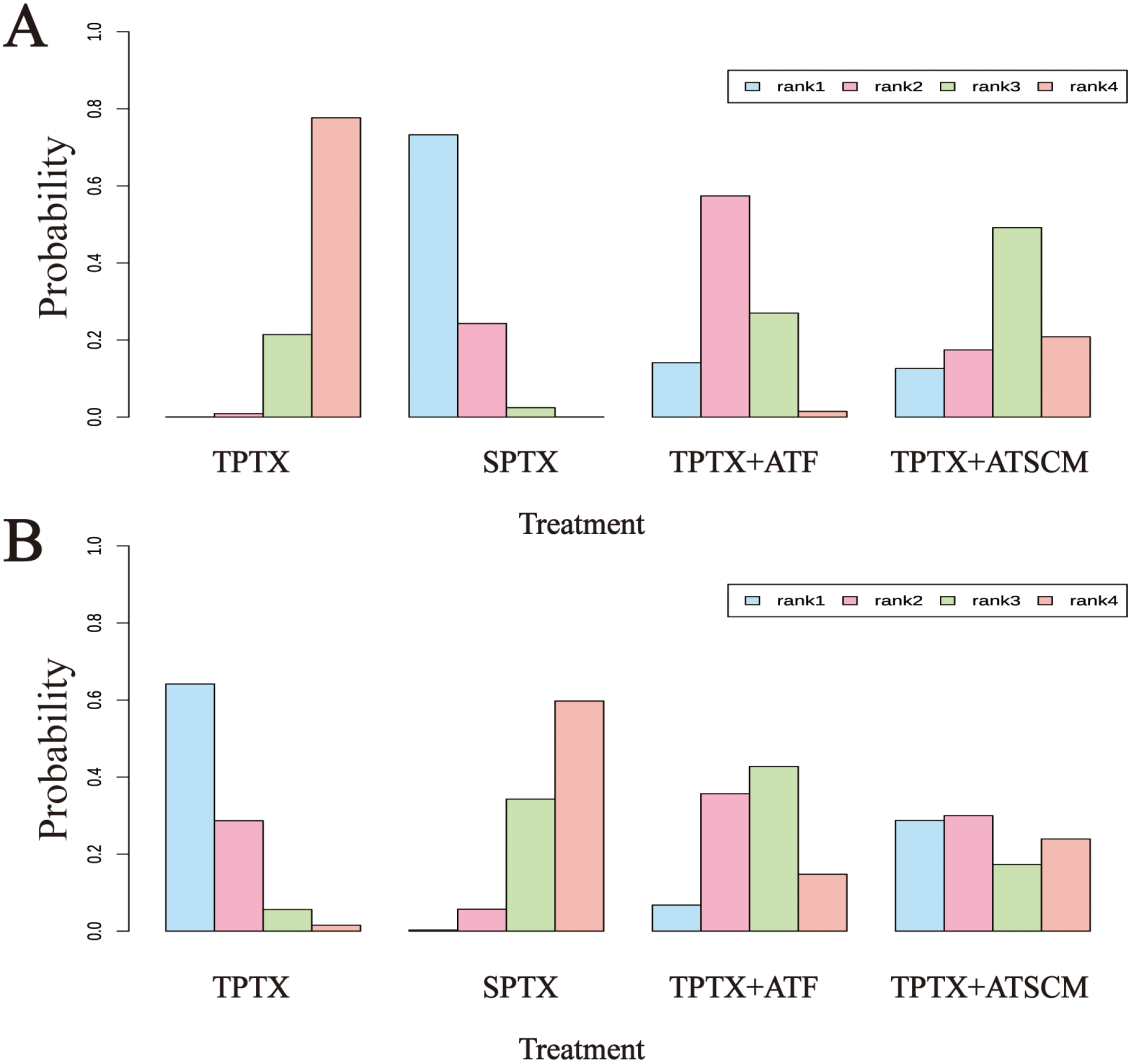
Values of the different rankings of the treatment strategies. **(A)** Recurrence; **(B)** Postoperative hypocalcemia.

**Figure 8:**
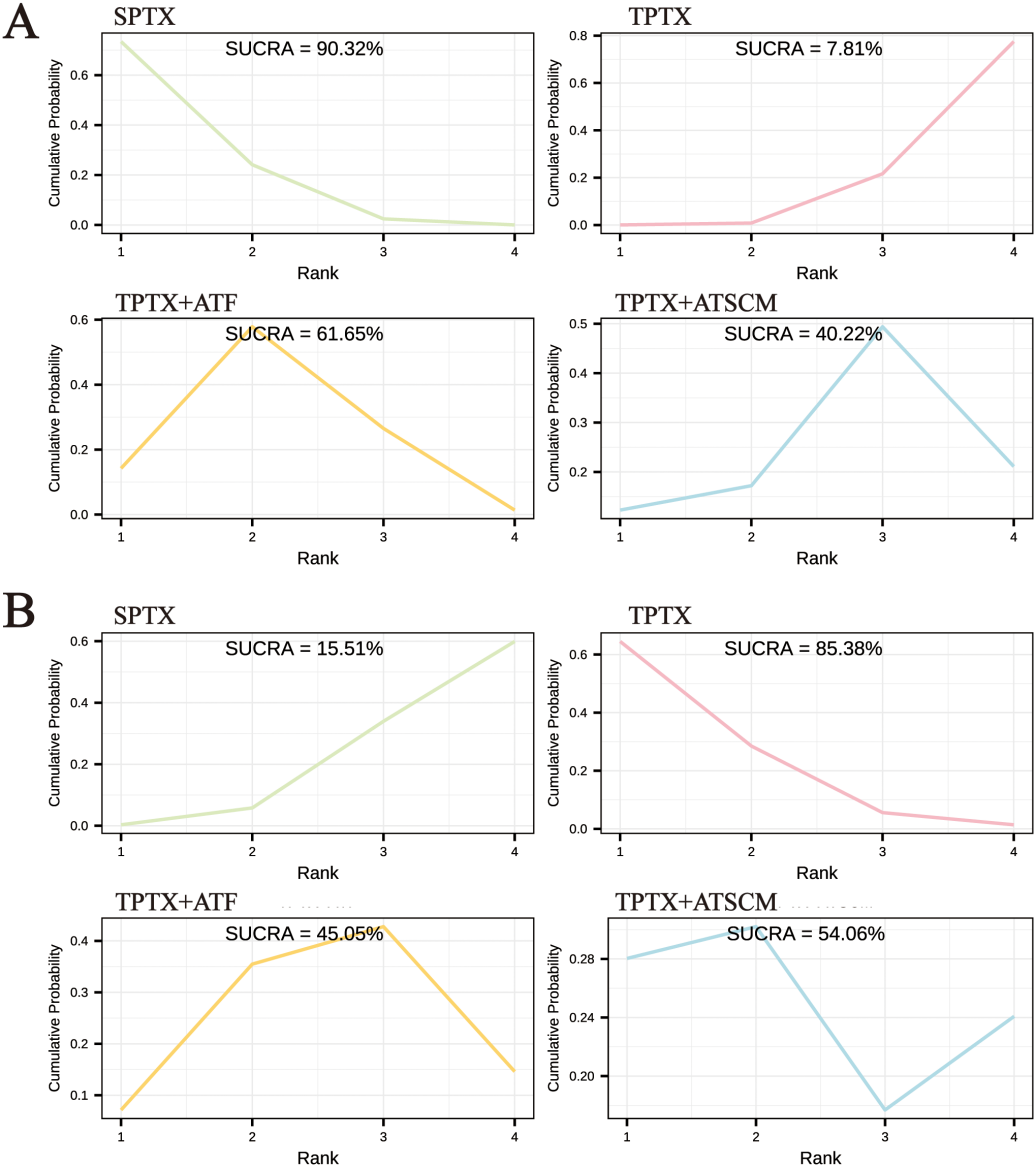
Cumulative ranking probability plot. **(A)** SUCRA Ranking Plots for recurrence; **(B)** SUCRA Ranking Plots for postoperative hypocalcemia.

We then ranked the procedures according to their probability of postoperative recurrence. The Rankogram (**Figure 7A**) illustrates the potential distribution of rankings for the four different surgical procedures concerning postoperative recurrence. The rankogram suggested that SPTX had the highest probability of ranking worst for recurrence, whereas TPTX had the highest probability of ranking best. TPTX + ATSCM appeared to rank more favorably than TPTX + ATF in terms of recurrence. However, this ranking-based observation should not be interpreted as evidence of a statistically significant difference between the two autotransplantation sites. To further summarize these conclusions, we calculated the SUCRA values (**Figure 8A**). The SUCRA values were consistent with the observations from the Rankogram. SPTX demonstrated the highest SUCRA value (90.26%), whereas TPTX maintained the lowest (7.75%). The overall risk level for postoperative recurrence among the two TPTX + AT procedures (TPTX + ATF and TPTX + ATSCM) was situated between SPTX and TPTX. The SUCRA value for TPTX + ATSCM (40.59%) was lower than that of TPTX + ATF (61.40%). However, SUCRA values should be interpreted as supportive ranking information rather than definitive evidence of a statistically significant difference between TPTX + ATSCM and TPTX + ATF.

A total of 15 studies, comprising 1,217 participants, were included in the analysis of postoperative hypocalcemia. SPTX was associated with a significantly lower risk of postoperative hypocalcemia compared with TPTX (OR 0.38, 95% CrI 0.11–0.99). Conversely, TPTX + ATF and TPTX + ATSCM showed a trend toward postoperative hypocalcemia compared to SPTX (OR 1.38, 95% CrI 0.67*–*3.23; OR 1.64, 95% CrI 0.32*–*7.65), and TPTX + ATSCM versus TPTX + ATF (OR 1.18, 95% CrI 0.18*–*6.23), but no statistically significant differences were observed (**Figure 6B**).

A postoperative hypocalcemia of recurrence was performed for the different surgical procedures for SHPT (**Figure 7B**). The results showed that TPTX had the highest probability of ranking first, suggesting the highest risk of postoperative hypocalcemia, while SPTX is most likely to rank last, reflecting the lowest probability of this complication. Notably, among the two procedures involving autotransplantation, TPTX + ATSCM exhibited a higher probability of being ranked worse than TPTX + ATF, suggesting that TPTX + ATF may carry a relatively lower risk of postoperative hypocalcemia. Subsequently, we calculated the SUCRA values, and these results further supported the findings from the probability ranking plot. TPTX demonstrated the highest SUCRA value for postoperative hypocalcemia (85.65%), followed by TPTX + ATSCM (53.76%) and TPTX + ATF (45.09%), while SPTX showed the lowest cumulative probability (15.51%). Overall, the Rankogram and SUCRA rankings consistently show that SPTX is most likely associated with the lowest risk of postoperative hypocalcemia, followed in increasing order of probability by TPTX + ATSCM, TPTX + ATF, and TPTX (**Figure 8B**).

## 4 Discussion

In this study, we used bibliometric analysis and Bayesian network meta-analysis to examine the major surgical strategies for SHPT, with particular focus on the choice of autotransplantation site after total parathyroidectomy. The principal findings were that TPTX was associated with a lower risk of recurrence than SPTX and TPTX + ATF, whereas no statistically significant differences were observed between forearm and sternocleidomastoid autotransplantation in terms of recurrence or postoperative hypocalcemia. These results indicate that, although implantation site remains an important practical consideration, current comparative evidence does not establish a clear advantage for either site. Importantly, the absence of statistically significant differences should not be interpreted as evidence of equivalence between the two implantation sites. Interpretation should nevertheless remain cautious because of the limited number of direct comparisons and the marked imbalance in sample size between the two autotransplantation groups.

Although calcimimetics have improved the medical treatment of SHPT, surgery remains necessary in a considerable number of patients with refractory disease. Even when the initial success of PTX is satisfactory, recurrence continues to represent an important long-term clinical concern. Our network meta-analysis demonstrated that the probability of recurrence with TPTX was significantly lower than with SPTX and TPTX + ATF. Furthermore, no statistically significant difference in postoperative recurrence was observed between TPTX + ATF and TPTX + ATSCM. Despite the lack of a statistically significant difference in direct comparison between these two autotransplantation sites, the forearm graft offers potential convenience in terms of postoperative monitoring and subsequent re-operation for recurrence. Forearm grafting may facilitate localization of graft-dependent recurrence by allowing comparison of intact PTH levels between the grafted and non-grafted arms, which can provide supportive information for subsequent therapeutic decisions (48). In contrast, sternocleidomastoid grafts may be less accessible for postoperative assessment because they are located within the cervical operative field. Furthermore, hyperplastic autografted parathyroid tissue located in the forearm can often be readily excised under local anesthesia (49). In contrast, the excision of hyperplastic autografted parathyroid tissue from the sternocleidomastoid muscle necessitates surgical intervention under general anesthesia (50). Furthermore, when the graft is placed in the forearm, the transplanted tissue is situated beneath the muscle fascia and maintains clear boundaries from surrounding vital neurovascular structures (such as the radial and ulnar nerves). This demarcation facilitates the relatively easy localization and complete excision of recurrent nodules (51). In contrast, the difficulty of removing hyperfunctioning parathyroid tissue from the cervical region depends largely on the source and location of recurrence. When recurrence is related to residual *in situ* parathyroid tissue after SPTX or missed cervical glands, re-exploration of a previously operated cervical field may be required, and scar formation together with altered anatomical planes may increase the technical difficulty of identifying and safely removing the recurrent tissue (52). However, graft-directed excision of parathyroid tissue implanted in the sternocleidomastoid muscle is anatomically distinct from deep cervical re-exploration, because this procedure is usually performed within the muscular implantation site and does not routinely require dissection at the level of the recurrent laryngeal nerve. Although no statistically significant difference in recurrence was observed between TPTX + ATF and TPTX + ATSCM, forearm grafting may offer practical advantages for postoperative monitoring and, when necessary, graft excision. While these considerations do not constitute evidence that forearm implantation confers superior recurrence outcomes, they may offer some reference for surgical decision-making.

Given that it severely impacts the quality of life for patients, postoperative hypocalcemia has garnered significant attention from numerous researchers (53). Consequently, the surgical approach for SHPT must seek a balance between preventing recurrence and mitigating the risk of permanent postoperative hypocalcemia (54). Given that postoperative hypocalcemia can substantially affect patient recovery and quality of life, it remains a key consideration when selecting a surgical strategy for SHPT. In our analysis, TPTX was associated with a significantly higher risk of postoperative hypocalcemia than SPTX. For postoperative hypocalcemia, no statistically significant difference was observed between TPTX + ATF and TPTX + ATSCM. Although ranking analyses suggested a possible trend favoring forearm implantation, this finding should be interpreted cautiously and does not establish a clinically meaningful difference in safety between the two approaches. From a practical perspective, differences in postoperative management may still influence procedural choice in some centers, but these considerations should be distinguished from evidence of superior clinical outcomes.

Operative time was inconsistently reported across the included studies, and the available data were insufficient for reliable quantitative comparison across surgical strategies. Operative duration should not be interpreted solely according to the extent of parathyroid gland removal, because SPTX may require careful preservation of the remnant gland and its vascular supply. In procedures involving autotransplantation, graft confirmation, graft preparation, and implantation-site preparation may further affect operative time. Forearm autotransplantation may require additional time due to the need for a second operative field, whereas sternocleidomastoid autotransplantation can be completed within the cervical incision. Similarly, although some studies reported a shorter median length of stay for TPTX + ATSCM than for TPTX + ATF, this difference cannot be attributed simply to forearm suture removal, since patients with a forearm incision generally do not need to remain hospitalized until suture removal in the absence of early local complications (21). Length of stay may also be affected by postoperative calcium monitoring, hungry bone syndrome, dialysis scheduling, institutional discharge criteria, comorbidities, and healthcare-system factors. Thus, although forearm implantation may offer practical advantages in postoperative monitoring and potential reintervention, it may also be associated with a greater short-term perioperative burden. These findings suggest that implantation-site selection should be based on a balanced consideration of long-term graft accessibility and early postoperative recovery, rather than an assumed overall advantage of one approach.

Several limitations should be considered. First, important clinical variables, including surgical indications, postoperative calcium, vitamin D supplementation requirements, and details regarding concomitant thymectomy, were not consistently reported across the included studies and therefore could not be reliably synthesized. Limited sample size and incomplete reporting also precluded analysis of several secondary outcomes, including surgical complications, mortality, and symptom relief. Second, recurrence and postoperative hypocalcemia were not uniformly defined across studies, which may have introduced outcome heterogeneity. Moreover, postoperative hypocalcemia was reported in only 15 of the 27 included studies, limiting the robustness of this analysis. Third, the limited number of direct head-to-head comparisons between the two autotransplantation approaches likely reduced statistical power and increased uncertainty in the comparative estimates. Finally, the predominance of retrospective cohort studies raises the possibility of selection bias, residual confounding, and other unmeasured sources of heterogeneity. In addition, practical surgical factors such as graft localization methods, reoperation difficulty, operative time, wound-related complications, and postoperative management protocols were not consistently reported, limiting our ability to determine whether proposed technical advantages of either implantation site translate into measurable clinical benefit.

## 5 Conclusion

In conclusion, both TPTX + ATF and TPTX + ATSCM are effective surgical strategies for SHPT. Based on the current evidence, no definitive difference was demonstrated between the two implantation sites with respect to recurrence or postoperative hypocalcemia. Forearm implantation may offer practical advantages for graft monitoring and possible reintervention in selected cases, but these considerations do not establish its superiority in clinical outcomes. Higher-quality comparative studies, particularly well-designed prospective trials, are still needed before any clear recommendation regarding the preferred implantation site can be made.

## Data Availability

The datasets analysed, and the analytic methods used during the current study are available from the corresponding author upon reasonable request.
