# Supplementary material for: Forearm vs sternocleidomastoid autotransplantation after total parathyroidectomy for secondary hyperparathyroidism: a network meta-analysis and global bibliometric study
Source: Front Endocrinol (Lausanne). 2026 Jul 22;17:1855948. doi: 10.3389/fendo.2026.1855948 (PMC13437453; doi:10.3389/fendo.2026.1855948)
Supplement: Supplementary file 1 [file Table1.docx]

**Supplementary Material 1: Bibliometric Search Strategy**

(TI=( Hyperparathyroidism, Secondary OR Secondary Hyperparathyroidism* OR secondary HPT OR SHPT OR renal hyperparathyroidism* OR renal secondary OR hyperparathyroidism* OR uremic hyperparathyroidism* OR hyperparathyroidism of renal disease*) OR AK=( Hyperparathyroidism, Secondary OR Secondary Hyperparathyroidism* OR secondary HPT OR SHPT OR renal hyperparathyroidism* OR renal secondary OR hyperparathyroidism* OR uremic hyperparathyroidism* OR hyperparathyroidism of renal disease*) OR AB=( Hyperparathyroidism, Secondary OR Secondary Hyperparathyroidism* OR secondary HPT OR SHPT OR renal hyperparathyroidism* OR renal secondary OR hyperparathyroidism* OR uremic hyperparathyroidism* OR hyperparathyroidism of renal disease*)) AND (TI=( Transplantation, Autologous OR autotransplantation* OR autograft* OR autologous transplantation* OR autotransplant* OR reimplantation * OR heterotopic transplantation * OR transplantation* OR parathyroid autotransplantation * OR parathyroid autograft* OR parathyroid reimplantation*) OR AK=( Transplantation, Autologous OR autotransplantation* OR autograft* OR autologous transplantation* OR autotransplant* OR reimplantation * OR heterotopic transplantation * OR transplantation* OR parathyroid autotransplantation * OR parathyroid autograft* OR parathyroid reimplantation*) OR AB=( Transplantation, Autologous OR autotransplantation* OR autograft* OR autologous transplantation* OR autotransplant* OR reimplantation * OR heterotopic transplantation * OR transplantation* OR parathyroid autotransplantation * OR parathyroid autograft* OR parathyroid reimplantation*))
