# Supplementary material for: Forearm vs sternocleidomastoid autotransplantation after total parathyroidectomy for secondary hyperparathyroidism: a network meta-analysis and global bibliometric study
Source: Front Endocrinol (Lausanne). 2026 Jul 22;17:1855948. doi: 10.3389/fendo.2026.1855948 (PMC13437453; doi:10.3389/fendo.2026.1855948)
Supplement: Supplementary file 3 [file Table3.docx]

**Supplementary Material 3: Search algorithm terms**

(1) “Hyperparathyroidism, Secondary” OR “Secondary Hyperparathyroidism” OR “secondary HPT” OR “SHPT” OR “renal hyperparathyroidism” OR “renal secondary hyperparathyroidism” OR “uremic hyperparathyroidism” OR “hyperparathyroidism of renal disease” (2) ”autotransplantation” OR ”autograft” OR ”Transplantation, Autologous autograft” OR ”autologous transplantation autograft” OR ”autotransplant autograft” OR ”reimplantation autograft” OR ”heterotopic transplantation autograft” OR ”transplantation autograft” OR ”parathyroid autotransplantation autograft” OR ”parathyroid autograft autograft” OR ”parathyroid reimplantation autograft” OR "Transplantation, Autologous" (3) “Recurrence” OR “Relapse” OR Recrudescence”.
